# Clinical outcome of admitted HIV/AIDS patients in Ethiopian tertiary care settings: A prospective cohort study

**DOI:** 10.1371/journal.pone.0226683

**Published:** 2019-12-30

**Authors:** Getandale Zeleke Negera, Teshale Ayele Mega

**Affiliations:** Department of Clinical Pharmacy, School of Pharmacy, Jimma University, Jimma, Ethiopia; Julius Centre, University Medical Centre, Utrecht, NETHERLANDS

## Abstract

**Background:**

Acquired ImmunoDeficiency Syndrome (AIDS) related illnesses are the leading cause of death in the developing world. However; there is limited evidence regarding the incidence of mortality among admitted HIV patients in Ethiopia.

**Objective:**

To determine the incidence of mortality and its predictors among admitted HIV/AIDS patients in selected tertiary care hospitals in Ethiopia.

**Methods:**

A prospective cohort study involving 136 admitted HIV/AIDS patients from April 1 to August 31, 2018 was conducted in selected tertiary care hospitals in Ethiopia. Data were collected on socio-demographic, clinical characteristics, and drug related variables. Kaplan-Meier and Cox regression were used to compare survival experience of the patients and identify independent predictors of mortality. Hazard ratio was used as a measure of strength of association and p-value of <0.05 was considered to declare statistical significance.

**Results:**

Of 136 patients, 80 (58.8%) were females. The overall in-hospital incidence of mortality was 2.83 per 1000 person-years. The incidences of mortality due to AIDS and non-AIDS related admissions were 6.1 [3.95, 8.67] and 5.3 [3.35, 8.23] per 1000 person-years respectively. The mean ± SD survival times among patients with AIDS and non-AIDS related illnesses were 32 ± 3.1 and 34 ± 3.3 days respectively (log rank p = 0.599). Being on non-invasive ventilation (AHR: 2.99, 95%CI; [1.24, 7.28]; p = 0.015) and having baseline body mass index (BMI) of less than 18.5 (AHR: 2.6, 95%CI; [1.03, 6.45]; p = 0.04) were independent predictors of mortality.

**Conclusion:**

The study found high incidence of in-hospital mortality among admitted HIV/AIDS patients in Ethiopian tertiary care hospitals. Being on non-invasive ventilation and body mass index (BMI) of less than 18.5 were found to be independent predictors of mortality.

## Back ground

Since the start of HIV epidemic, an estimated 77.3 million people had been infected and 35.4 million have died of AIDS-related illnesses, globally [1]. In sub-Saharan African, about 24.7 million people are living with the virus, making it the most affected region in the world. This region covers 74% of HIV-related deaths [2]. Ethiopia has the largest populations of HIV infected people in the region. According to an estimate by the Federal HIV/AIDS Prevention and Control Office (FHAPCO), there are over 738,976 people living with HIV/AIDS in Ethiopia [3].

AIDS-related illnesses are responsible for most of the morbidities and mortalities in HIV infected persons. In 2016 alone, 1 million people died of AIDS-related illnesses. Since the widespread use of combined antiretroviral therapy (cART) in the mid-1990s, a significant reduction in AIDS-related mortality was observed [4].

In the late cART era, hospitalization rates have decreased, mostly due to a decrease in the rate of AIDS-related illnesses. Consequently, non-AIDS related illnesses have become more common as the cause of mortality and hospitalization [5–8]. This shift in the cause of death and hospitalization is associated with the widespread use of combined antiretroviral therapy (cART) and better care in developed settings [9–11].

Contrarily, observational studies from Sub-Saharan Africa indicated, in-hospital mortality of HIV-infected individuals remained higher [12–14]. According to recent study from South Africa, in-hospital mortality was reported to be as high as 38.9% and AIDS-related illnesses accounted for majority of the death [15]. Similarly, multicenter prospective cohort study conducted in West Africa found AIDS-related illnesses as a major cause of death [14].

In Ethiopia, despite free cART program, HIV/AIDS is still responsible for considerable amount of hospitalization with an overall bed occupancy rate of 18.9% [16]. Although the mortality rate of HIV patients in Ethiopia was reported to be as high as 44.5% in some hospitals, there is limited study regarding mortality in admitted HIV/AIDS patients [17]. Therefore, the aim of this study was to determine in-hospital incidence of mortality and its predictors among admitted people living with HIV (PLWHA).

## Methods

### Study design and setting

A prospective cohort study was conducted among 136 hospitalized HIV/AIDS patients admitted to Tikur Anbessa Specialized Hospital (TASH) and Jimma University Medical Center (JUMC). Data were collected prospectively from April 1, to August 31, 2018. TASH is the largest referral hospital in the country, with 700 beds. It is located in Addis Ababa, the capital of Ethiopia. It is now the main teaching hospital for both clinical and preclinical training of most disciplines. It is also an institution where specialized clinical services that are not available in other public or private institutions are rendered to the whole nation. On the other hand, JUMC is located in Jimma town, 355 km from Addis Ababa. It is currently the only teaching and specialized hospital in the southwest region of Ethiopia. The hospital serves as a referral site and provides specialized care for southwest Ethiopia with a catchment population of about 15 million. The available diagnostic tools used for this study in the settings includes; Human immunodeficiency virus (HIV) test was performed using the HIV 1/2 STAT-PAK1 RDT (Chembio Diagnostics, Medford, NY, USA). Complete blood counts were performed using hematologic analyzers: XT-1800i (Sysmex, Japan), KX-21 N^™^ (Sysmex, Japan), and Cell-Dyn 18001 (Abbot, USA). Mycobacterium tuberculosis diagnosis was made using Cepheid Xpert MTB/RIF^®^). Test for Hepatitis B surface antigen (HBsAg) was done using the rapid diagnostic tests Determine^™^ (Alere, Waltham, MA, USA), and Bio-Dignos HBsAg cassette (Nantong Diagnose, China); hepatitis C virus (HCV) antibody (anti-HCV) test was done using the rapid diagnostic test EGENS HCV Test cassette (Jiangsu, China), and SD BIOLINE HCV (Standard Diagnostics, Yongin-si, Republic of Korea). Stroke and heart failure diagnosis was made using computed tomography CT-Scan, S192 from Optima CT/GE Healthcare and Echocardiography (HUMANSCAN Co Ltd) respectively. Weight and height were measured using WS703 from Narang medical limited.

### Study population and variables

We included all adult (age>18years) HIV/AIDS patients, admitted to medical, intensive care unit (ICU) and surgical wards for at least 24 hours. Signed informed consent was taken from all patients included in the study. Patients with readmission, incomplete medical records, pregnant and obstetric admissions were excluded. Data were collected on socio-demographic characteristics (sex, age, ethnicity, marital status, area of residence, educational, and occupational status, monthly income, body mass index (BMI)), Diseases Related Factors (reason for admission, time of HIV diagnosis, history of OI, current CD4 count, WHO clinical stage, co-morbidities), Treatment Related Factors (OI prophylaxis (Cotrimoxazole prevention therapy (CPT)), non-invasive ventilation, cART status, duration on cART, type of cART regimen) and clinical outcome (mortality). Monthly income of the patients was measured using Ethiopian birr (ETB) and it’s classified based on Ethiopian Civil Service monthly salary scale for civil servants.

### Sample size determination and sampling technique

The sample size was calculated using a single population proportion formula, $\frac{No=n(1-P){PZ}^{2}}{d^{2}}$ where, No = minimal sample size required, z = 1.96 (normal deviate corresponding to 95% confidence interval), d = 0.05 (degree of precision of 5%), p = 0.445 (a mortality rate of 44.5%; from study conducted in Addis Ababa [17]). Thus, $\frac{No=0.445*1.96^{2}(1-0.445)}{0.05^{2}}=379$ patients. Since the target population is less than 10,000 using correction formula nf = N_o_/1+N_o_/N. Where nf is the corrected sample size and N is obtained from the last 5 months admission of patients in the two hospitals, which was 185. The corrected sample size is nf = 379/1+379/185 = 124. By adding 5% contingency value, the final sample size was 131 patients. Based on the previous admissions, this number is proportionally divided for both hospitals in the ratio of 1:1.2. Accordingly, 71 and 60 patients were allocated for JUMC and TASH, respectively.

### Data collection procedure and quality assurance

Clinical data were collected prospectively from active patient follow up chart using English version checklists. The data collection tool was carefully prepared after reviewing relevant literatures to enable the data collectors to gather all the information required to address the study objectives. Pre-test was conducted on 5% of patients. A 2-day training was provided on the data collection tool and the general procedures for data collectors i.e. 3 pharmacists (B.Pharm) and 3 nurses, and 2 medical interns who were acting as supervisor.

#### Patient follow-up and outcome measurement

The follow-up started at hospital admission and ended if patients were discharged with improvement or developed an outcome. In-hospital incidence of mortality was the clinical outcome of this study. The underlying cause of death was extracted from the death summary (possible cause of death) as determined by the attending physicians. Subjects were considered as censored if: they do not develop the event (death) during study period or lost on follow up.

### Ethical consideration

The study was approved by Institutional Review Board (IRB) of Jimma University. It has designated with an IRB number of IHRPGB/219/2018. The principal investigator or data collectors briefed the aim of the study to the patients and signed informed consent was taken from all participants or their care givers prior to data collection. During data collection, confidentiality was ensured and for this reason, name and address of the patient was not recorded in the data collection check list.

### Statistical analysis

Data were entered into EpiData 3.2 and exported to statistical package for social sciences (SPSS) Version 21.0 for cleaning and analysis. Descriptive analysis was performed and results were presented by text, tables and figure. Kaplan-Meier (log rank test) was used to compare survival experience of the patients. Chi-square test was performed to check adequacy of cells before performing Cox regression. Bivariate Cox regression was performed to identify candidate variables for multivariable Cox regressions. Variables with p-value ≤ 0.25 in bivariate regression were considered as candidates for multivariable regression. Multivariable Cox regression was performed to identify independent predictors of in-hospital mortality. Hazard ratio was used as a measure of strength of association. Predictors with p-value < 0.05 were considered to declare a statistical significance.

## Results

### Overview of the study participants

During the study period, 144 HIV-positive patients were admitted to JUMC and TASH. Of these, 8 patients were excluded (age <18 = 6, readmitted = 2). During the follow up period six patients were lost to follow up, basically due to loss of hope in modern medicine and strong belief in spiritual healing. Finally, 136 patients were included in the data analysis. Majority, 76 (55.9%) of the patients were from JUMC. The overall analysis time under risk was 74.9 months or 6.24 years.

#### Socio-demographic and behavioural characteristics

Of 136 eligible patients, 80 (58.8%) of them were females. The mean ± SD age of the patients was 36.8 ±11.3 years. More than half, 73 (53.7%) of the patients, had baseline body mass index (BMI) of greater than 18.5 kg/m2. Majority, 99 (72.8%) of the patients were from urban areas. Fifty one (37.5%) of them had attended primary education and only 36 (26.5%) of them had higher education. A higher proportion of the patients, 69 (50.7%) were unemployed. Regarding the marital status, 58 (42.64%) of the patients were married while, 22 (16.2%) of them were divorced. Fifty nine (43.4%) of the patients were from Oromo ethnic group followed by Amhara 43 (31.6%). With respect to social drug use habit, 34 (25%) and 14 (10.3%) of the patients reported alcohol consumption and cigarette smoking respectively. About 56 (41.2%) participants had average monthly income of less than 1500 Ethiopian Birr (ETB). Socio-demographic and behavioral characteristics are depicted in Table 1.

**Table 1:** Socio-demographic and behavioral characteristics of the study cohort at JUMC and TASH, from April 1 to August 31, 2018.

Variables		Death		Total n = 136	P-value
		Yes	No		
Study site	JUMC	20 (26.3)	56 (73.7)	76 (58.8)	0.56
	TASH	19 (31.7)	41 (68.3)	60 (41.2)	
Age, mean(± SD)		39.7 (±13.1)	35.6 (±10.4)	36.8±11.3	0.14
Sex	Female	22 (27.5)	58 (72.5)	80 (58.8)	0.71
	Male	17 (30.4)	39 (69.6)	56 (41.2)	
BMI (Kg/m^2^	<18.5	20 (31.7)	43 (68.3)	63 (46.3)	0.46
	>18.5	19 (26)	54 (74)	73 (53.7)	
Residence	Urban	25 (25.3)	74 (74.7)	99 (72.8)	0.15
	Rural	14 (37.8)	23 (62.2)	37 (27.2)	
Educational level	No formal Education	22 (75.9)	7 (24.1)	29 (21.3)	0.77
	Primary	16 (31.4)	35 (68.6)	51 (36.7)	
	Secondary	7 (35)	13 (65)	20 (14.7)	
	Higher Education	27 (75)	9 (25)	36 (27.3)	
Occupation	Employed	16 (23.9)	29 (76.1)	67 (49.3)	0.22
	Unemployed	23 (33.3)	46 (66.7)	69 (50.7)	
Marital status	Married	13 (22.4)	45 (77.6)	58 (42.6)	0.26
	Single	11 (34.4)	21 (65.6)	32 (23.5)	
	Divorced	10 (41.7)	14 (58.3)	24 (17.6)	
	Widowed	5 (22.7)	17 (77.3)	22 (16.3)	
Ethnicity	Oromo	17 (28.8)	42 (71.2)	59 (43.4)	0.94
	Amhara	13 (30.2)	30 (69.8)	43 (31.6)	
	Others*	9 (26.5)	25 (73.5)	34 (25)	
Alcohol	Drinker	13 (38.2)	21 (61.8)	34 (25)	0.15
	Non drinker	26 (25.5)	76 (74.5)	102 (75)	
Smoking	Smoker	6 (42.9)	8 (57.1)	14(10.3)	0.21
	Non-smoker	33 (27)	89 (73)	122 (89.7)	
**Monthly income (ETB)	(<1500)	17 (30.4)	39 (69.6)	56(45.5)	0.53
	(1500–3000)	13 (33.3)	26 (66.7)	39 (31.7)	
	(3000–5000)	2 (15.4)	11 (84.6)	13 (9.6)	
	(>5000)	3 (20)	12 (80)	15 (12.2)	

* Tigre, Gurage, Kafa, Nuer.

** Based on Ethiopian Civil Service monthly salary scale for civil servants. ETB: Ethiopian birr, BMI: body mass index, SD: standard deviation.

### Baseline clinical, laboratory and drug related characteristics

A CD4 T lymphocytes (CD4 cells) count was done in 133 (97.8%) patients, and 80 (60.1%) of them had less than 200cells/μL. The mean ± SD hemoglobin (g/dl) of the patients was 10.3 ± 3.9. The serologic test showed that, 13 (9.6%) and 9 (6.6%) of the patients had positive hepatitis B virus (HBV) and hepatitis C virus (HCV) surface antigen respectively. Majority, 107 (78.7%) of the patients were already on cART. Of patients on cART, 93 (86.9%) of them were on treatment for more than 6 months. Majority, 87 (81.3%) of the patients were on first line cART regimen and a combination of Tenofovir, Lamivudine with Efavirenz (TDF+3TC+EFV) was the most, 77 (56.6%) prescribed cART regimen.

Regarding WHO clinical stage, 60 (44.1%) of the patients were at WHO clinical stage four. In-hospital survival was found to have a significant association with WHO clinical stage of the patients (p = 0.04). The mean (±SD) since the diagnosis of HIV was 4.5± 4.1 and 6.4± 5.2 years for survivors and non-survivors respectively (p = 0.01). Majority, 108 (79.4%) of the patients were on cotrimoxazole preventive therapy (CPT) at admission. About, 59 (43.4%) of the patients had previous history of opportunistic infections. Baseline clinical, laboratory and drug related characteristics of the study subjects are shown in Table 2.

**Table 2:** Baseline clinical, laboratory and drug related characteristics of the patients at JUMC and TASH, from April 1 to August 31, 2018.

Variable		Death		Total	P value
		Yes	No		
Current CD4 cells/μL	≤200	26 (32.5)	54 (67.5)	80 (60.1)	0.22
	>200	12 (22.6)	41 (77.4)	53 (39.9)	
Haemoglobin(g/dl), mean ± SD		10.5±3.5	10.3±4.2	10.3± 3.9	0.45
HBV status	Not known	16 (29.1)	39 (70.9)	56 (41.2)	0.7
	Negative	18 (26.9)	49 (73.1)	67 (49.3)	
	Positive	5 (38.5)	8 (61.5)	13 (9.5)	
HCV status	Not known	15 (28.3)	38 (71.7)	54 (39.7)	0.56
	Negative	20 (27.4)	53 (72.6)	73 (53.7)	
	Positive	4 (44.4)	5 (55.6)	9 (6.6)	
cART status	Yes	26 (24.3)	81 (75.7)	107 (78.7)	0.03
	No	13 (44.8)	16 (55.2)	29 (21.3)	
cART regimen	TDF+3TC+EFV	13 (16.9)	64 (83.1)	77 (72)	0.06
	AZT+3TC+NVP	4 (40)	6 (60)	10 (9.3)	
	Others*	9(19.5)	11(18)	20 (18.7)	
Duration on cART	<6 months	0 (0)	14 (100)	14 (13.1)	0.02
	>6 months	26 (28)	67 (72)	93 (86.9)	
WHO clinical stage	I	2 (11.8)	15(88.2)	17 (12.5)	0.04
	II	7 (28)	18 (72)	25 (18.4)	
	III	6 (17.6)	28 (82.4)	34 (25)	
	IV	24 (40)	36 (60)	60 (44.1)	
HIV sero-status	Known RVI patient	29 (26.1)	82 (73.9)	111 (81.6)	0.17
	Newly diagnosed	10 (40)	15 (60)	25 (18.4)	
Time since diagnosis in years (mean ±SD)		6.4±5.2	4.5±4.1	5.1 ± 4.5	0.01
Prophylaxis(CPT)	Yes	26 (24.1)	82 (75.9)	108 (79.4)	0.02
	No	13 (46.4)	15 (53.6)	28 (20.6)	
Co-morbidity	Yes	19 (38.8)	30 (61.2)	49 (36)	0.05
	No	20 (23)	67 (77)	87 (74)	
History of OIs	Yes	14 (23.7)	45 (76.3)	59 (43.4)	0.26
	No	25 (32.5)	52 (67.5)	77 (56.6)	

*AZT+3TC+ATV/R, TDF+3TC+NVP, AZT+3TC+EFV, TDF+3TC+ ATV/R, CD4: cluster of differentiation. HBV: hepatitis B virus, HCV: hepatitis C virus, cART: combined antiretroviral therapy, WHO, world health organization, RVI: retroviral infection, CPT: cotrimoxazole preventive therapy, OIs: opportunistic infections.

### Clinical outcome

#### Mortality

The overall in-hospital incidence of mortality was found to be 2.83 per 1000 person-years. The incidences of mortality were 6.1 [3.95, 8.67] and 5.3 [3.35, 8.23] per 1000 person-years for AIDS and non-AIDS related admissions respectively (p = 0.68). Diagnosis of AIDS and Non-AIDS illness was made based on annex “A” by the caring physician who is blind to this study. Twenty (28.6%) patients were died from JUMC, while nineteen (28.8%) died from TASH (p = 0.49). The diagnosis of HIV was made in 111 (81.6%) patients before current hospital admission and there was no baseline difference in case-fatality between known HIV/AIDS patients and those newly diagnosed at admission (p = 0.16).

A difference in the incidence of mortality was observed between those on cART and non-cART at baseline (p = 0.030). In-hospital incidence of mortality was also different at baseline between patients who were on CPT and non-CPT (p = 0.02). A higher proportion of patients who were on non-invasive ventilation were died (p<0.001). The mean ±SD, length of hospital stay was 16.5±10 and 16.7± 12.9 days for in-hospital survivors and non-survivors respectively (p = 0.01). Follow up outcome of the study subjects are shown in Table 3.

**Table 3:** Follow up outcome of hospitalized HIV/AIDS patients at JUMC and TASH, from April 1 to August 31, 2018.

Variables		Death			p-value
		Yes	No	Total (136)	
Study site	JUMC	20 (26.3)	56 (73.7)	76 (55.9)	0.49
	TASH	19 (31.6)	41 (68.4)	60 (44.1)	
Reason for admission	AIDS-Related	20 (30.3)	46 (69.7)	66 (48.5)	0.68
	Non-AIDS Related	19 (27.1)	51 (72.9)	70 (51.5)	
HIV sero-status	Known	29 (26.1)	82 (73.9)	111 (81.6)	0.16
	Newly diagnosed	10 (40)	15 (60)	25 (18.4)	
cART status	Yes	26 (24.3)	81 (75.7)	107 (78.7)	0.030
	No	13 (44.8)	16 (55.2)	29 (21.3)	
CPT status	Yes	26 (24)	82 (76)	108 (79.4)	0.02
	No	13 (46.4)	15 (53.6)	28 (20.6)	
Non-invasive ventilation	Yes	26 (54)	22 (46)	48 (35.3)	<0.001
	No	13 (14.8)	75 (85.2)	88 (64.7)	
Length of hospital stay (mean± SD)days		16.7± 12.9	16.5± 10	16.5±10.9	0.01

AIDS-related admissions were responsible for 20 (51.3%) of in-hospital death. The commonly reported cases of death among AIDS-related admissions were, cryptococcal meningitis, 5 (25%), disseminated tuberculosis, 4 (20%), and cerebral toxoplasmosis, 2 (10%).

Gastrointestinal bleeding, 7 (37.8%), non-recurrent bacterial infections, 6 (36%), and Non-AIDS defining cancers (NADC) were the major contributors for case fatality among non-AIDS related admissions. Cause of death among patients admitted with AIDS and non-AIDS related diseases are depicted in Table 4.

**Table 4:** Cause of death among HIV/AIDS patients admitted with AIDS and non-AIDS related diseases at JUMC and TASH, from April 1 to August 31, 2018.

**Cause of death among AIDS-related admissions**	1. Cryptococcal meningitis, 5 (25%)
	2. Disseminated Tuberculosis, 4 (20%)
	3. Cerebral toxoplasmosis, 2 (10%)
	4. Others*, 9 (45%)
**Cause of death among non-AIDS related admissions**	**1**. Gastrointestinal bleeding, 7 (36.8%)
	**2** Non-recurrent bacterial infections, 6 (31.6%)
	**3**. Non-AIDS defining cancers (NADC), 5 (26.3%)
	4. Others**, 6 (31.6%)

*Pneumocystis jiroveci Pneumonia, pulmonary tuberculosis, Aids Defining Cancers (ADC)

**Cardiovascular disease, Stroke, sepsis

**Fig 1:**
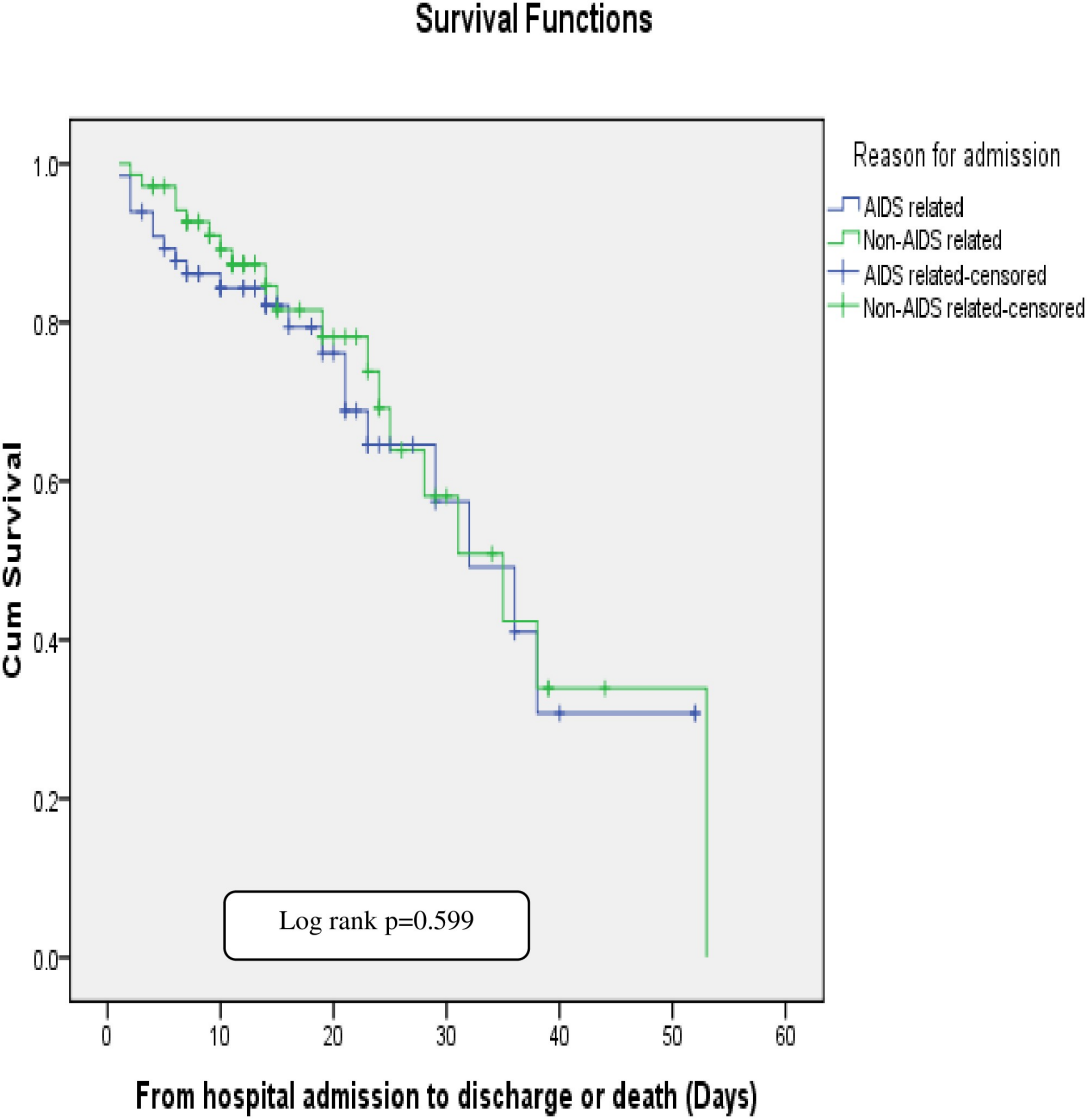
Survival estimates for patients with AIDS and non-AIDS related admissions at JUMC and TASH, from April 1 to August 31, 2018.

The mean ± SD survival times among patients with AIDS and non-AIDS related diseases were 32 ± 3.1and 34± 3.3 days respectively (log rank p = 0.599) (Fig 1).

#### Predictors of in-hospital mortality

Binary Cox regression was done to identify the association between baseline characteristics and death. Accordingly, sex, body mass index (BMI), residence, HIV sero-status, history of OI, current CD4+ count, reason for admission, WHO clinical stage, cotrimoxazole preventive therapy (CPT), non-invasive ventilation and co-morbidity had p-value of < 0.25. With a multivariate Cox regression modeling, being on non-invasive ventilation (AHR: 2.99; 95%CI: [1.24, 7.28]; p = 0.015) and body mass index (BMI) of less than 18.5 (AHR: 2.6; 95%CI: [1.03, 6.45]; p = 0.04) remained significant predictors of mortality. Table 5 shows Cox-proportional hazard regression analysis.

**Table 5:** Crude and adjusted Cox-proportional hazard regression for predictors of mortality at JUMC and TASH, from April 1 to August 31, 2018.

Variables		Death		*CHR [95%CI]	P-value	*AHR [95%CI]	P-value
		Yes	No				
Sex	Female	22(27.5)	58(72.5)	1			
	Male	17(30.4)	39(69.6)	1.57[0.82,3.01]	0.17	1.8[0.77,4.54]	0.16
BMI (Kg/m^2^)	>18.5	19(26)	54(74)	1			
	<18.5	20(31.7)	43(68.3)	1.5[0.79,2.90]	0.20	2.6[1.03,6.45]	0.04
Residence	Urban	25(25.2)	74(74.8)	1			
	Rural	14(37.8)	23(62.2)	2.0[1.03.3.90]	0.04	1.35[0.57,3.12]	0.49
WHO clinical stage	I	2(11.8)	15(88.2)	1			
	II	7(28)	18(72)	1.79[0.36.8.88]	0.47	1.1[0.16,8.01]	0.89
	III	6(17.6)	28(82.4)	1.22[0.25.6.09]	0.80	1.2[0.17,8.5]	0.85
	IV	24(40)	36(60)	3.5[0.84,15.05]	0.08	2.4[0.44,13.02]	0.31
Reason for Admission	Non- AIDS related	19(27.1)	51(72.9)	1			
	AIDS-related	20(30.3)	46(69.7)	1.18[0.63,2.24]	0.60	0.76[0.29,1.92]	0.56
CPT use	Yes	26(24)	82(76)	1			
	No	13(46.4)	15(53.6)	2.79[1.4,5.55]	0.003	1.4[0.34,5.81]	0.63
Co-morbidity	No	20(23)	67(77)	1			
	Yes	19(38.8)	30(61.2)	1.52[0.81,2.89]	0.19	1.6[0.68,3.77]	0.27
Non-invasive ventilation	No	13(14.8)	75(85.2)	1			
	Yes	26(54.2)	22(45.8)	3.03[1.55,5.95]	0.001	2.99[1.24,7.28]	0.015
History of OI	Yes	14(23.7)	45(76.3)	1			
	No	25(32.5)	52(67.5)	1.98[1.01,3.9]	0.048	2.3[0.86,6.04]	0.63
HIV serostatus	New	10(40)	15(60)	1			
	Known	29(26.1)	82(73.9)	2.2[1.06,4.55]	0.03	3.2[0.63,16.6]	0.15
Current CD4 count (mean±SD)				0.999[.998,1.0]	0.18	1.0[0.99,1.002]	0.95

*AHR: adjusted hazard ratio, CHR: crude hazard ration

## Discussion

This study assessed the clinical outcome of hospitalized HIV/AIDS patients treated at two selected Ethiopian tertiary care hospitals. The (mean ± SD) age of the patients was (36.8± 11.34) years. About 13 (9.6%) and 9 (6.6%) of them had HBV and HCV co-infection respectively. The overall in-hospital incidence of mortality was 2.83 per 1000 person-years. The incidences of mortality among patients with AIDS and non-AIDS related illnesses were 6.1 [3.95, 8.67] and 5.3 [3.35, 8.23] per 1000 person-yearsrespectively (p = 0.68). Most of the deaths were attributed to cryptococcal meningitis, 5 (25%), followed by disseminated tuberculosis, 4 (20%), and cerebral toxoplasmosis, 2 (10%). The mean ± SD survival times of the patients with AIDS and non-AIDS related diseases were 32 ± 3.1 and 34 ± 3.3 days respectively (log rank p = 0.599). Baseline body mass index (BMI) of less than 18.5 (p = 0.04) and being on non-invasive ventilation p = 0.015) were independent predictors of mortality.

The (mean ± SD) age of the admitted patients was (36.8± 11.34) years. This is similar with the study conducted in Uganda [18], but less than reported figures in the United States [19].

This finding indicated that HIV/AIDS is affecting the most productive age group of the society, having a significant impact on their family and country as a whole. About 13 (9.6%) and 9 (6.6%) of the patients had HBV and HCV co-infection respectively. This finding is comparable for HBV with CDC report, that 10% of HIV infected Americans are co-infected with HBV but lower than reported figure for HCV in which 25% of them are co-infected with HCV [20].

In this study, the over all in-hospital incidence of mortality was 2.83 per 1000 person-years. This finding is similar with the report from Nigeria (28.6%) [21]. However, in a finding from Ghana, higher (40.6%) in-hospital mortality was reported [22]. The deviation might be due to the study design (retrospective), larger sample size (547 vs. 136) and study setting. Slightly higher proportion of death was reported among patients with AIDS related illnesses, 51.3% versus 48.7% (*p = 0*.*68*). This finding is in line with the study conducted in Spain, in which 53% of deaths were attributed to AIDS defining illnesses [23]. Similar study from Brazil reported that in-hospital mortality was almost two times higher in AIDS-related hospitalizations than in non-AIDS-related hospitalizations [24]. However, several studies from developed countries reported non-AIDS-related illnesses as being the most common cause of death [25–27]. In a Swiss cohort, majority (84%) of deaths were caused by AIDS unrelated cases [26]. This difference could be because of better socio-economic, education and patient care in developed settings.

Cryptococcal meningitis (25%), disseminated tuberculosis (20%) and cerebral toxoplasmosis (10%) were the leading cause of death among AIDS-related admissions. This finding is comparable with the study conducted in West Africa [14]. The possible reason that cryptococcal meningitis was a leading cause of death is because of the fact that, majority of patients admitted with AIDS-related illnesses had a CD4 cell count of <200 cells/μL. In contrary to our finding, tuberculosis is the leading cause of morbidity and mortality among people living with HIV worldwide [20].

Among patients admitted with non-AIDS related illnesses, gastrointestinal bleeding (36.8%), non-recurrent bacterial infections (31.6%), and non-AIDS defining cancers (NADC) (26.3%) were the major cause of death. However, studies conducted in New York [28] and West Africa [14] reported sepsis as a major cause of death in non-AIDS related admissions. The mean ± SD survival time between those admitted with AIDS and non-AIDS related illnesses was not statistically different (log rank p = 0.599).

In this study, patients with low body mass index (<18.5kg/m2) were found to be at increased risk of death (AHR: 2.6[1.03, 6.45]). This finding is supported by the study conducted in Rwanda [29]. According to Anjali Sharma et.al [30], AIDS related admissions had doubled the hazard of death in underweight (BMI<18.5 kg/m2) patients (AHR: 2.04, 95% CI [1.03, 4.04]). Even though the underlying mechanisms placing underweight HIV-infected patients at risk of death are unclear, several factors which could have facilitated progression to death were more prevalent among our study participants. The most important once include, majority of patients at advanced stage of the disease and low CD4 count. Furthermore, malnutrition (typically defined as a body mass index (BMI) of <18.5 kg/m2) aggravates the underlying immunosuppression enhancing their susceptibility to various infections and HIV disease progression [31–32].

Being on non-invasive ventilation (NIV) was also associated with higher hazard of mortality (AHR: 2.99 [1.24, 7.28]). This finding is in line with studies conducted in Uganda [13], Mexico [33] and Brazil [34]. The possible explanation could be that most of patients in this study were at an advanced stage of the disease and they tended to be critically ill with multiorgan failure during admission.

Our study has several limitations. Inclusion of small number of patients; loss of follow-up during the study period; inclusion of only two tertiary hospitals and lack of diagnostic equipment, such as viral load measurement, were the limitations of the present study. We recommend a large scale, multicenter prospective cohort study to be conducted, including other tertiary care hospitals in the country.

## Conclusion

This study found high incidence of in-hospital mortality among admitted HIV/AIDS patients in Ethiopian tertiary care settings. The overall incidence of mortality in our study is still higher than middle and high income countries. Furthermore, this study found being on non-invasive ventilation and low body mass index as independent predictors of mortality. This target group’s needs special attention as they carried higher hazards of mortality.

## Supporting information

S1 Dataset Data collection tool. (DOCX)

S2 Dataset Clinical outcome of admitted HIV/AIDS patients. (SAV)

S3 Dataset List of AIDS-related illnesses. (DOCX)
