# Supplementary material for: Clinical outcome of admitted HIV/AIDS patients in Ethiopian tertiary care settings: A prospective cohort study
Source: PLoS One. 2019 Dec 30;14(12):e0226683. doi: 10.1371/journal.pone.0226683 (PMC6936777; doi:10.1371/journal.pone.0226683)
Supplement: S1 Dataset — (DOCX) [file pone.0226683.s001.docx]

**ANNEXES**

**ANNEX I: Informed consent**

**Consent form:** Hello, my name is Getandale Zeleke, a lecturer in Jimma University. We are conducting a study on “**Clinical outcome of admitted HIV/AIDS patients in Ethiopian tertiary care settings.”** I would like to interview you a few questions about you, your disease and treatment you are getting and your laboratory findings (from medical chart). The study will provide information that might enable the health personnel and the government to get an insight of outcomes of hospitalization among HIV/AIDS patients and its predictors. We would like to take your time to respond to our interview questions and it will take approximately15 minutes. We also request you to answer as truthfully as possible. Your answer will not be revealed to the health personnel or any other people, and the information you give will be treated anonymously and confidential. This research imposes no risk and therefore no compensation willbeprovidedfor yourparticipationinthisstudy.Yourparticipationistotallyvoluntaryand you can withdraw anytime or refuse to continue and this will not influence the way you are treated in the health institution or in thecommunity.


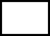

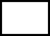
Are you willing to participate? **Yes No**

Signature of the participant

Day

Signature of data collector Day

Name of Principal Investigator: GetandaleZeleke Phone No:0912892539

[Email:Getandale@gmail.com](mailto:Getandale@gmail.com)

**ANNEX II: Data collection tool**

- 1. **Demographic, psychosocial and behavioral characteristics of thepatients CardNo.**


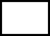
**Hospital site: JUMC
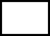
 TASH**

**Admission ward: internal medicine
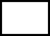
 ICU
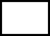
 surgery
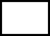
**

1. Sex Male
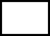
Female
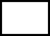

2. Age
3. BMI
4.
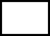
Educational level No formal education
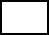
 Primary school [1-8]
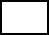
Secondary school [9-12]
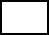
Higher education
5.
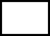
Area of residence: Urban
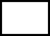
Rural
6. Ethnicity:Oromo
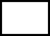
 Amara
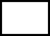
other
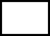
 specify__________
7. Risky behaviors :smoking status Smoker
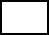
Non-smoker
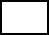


Alcoholic status: Drinker (drunk any quantity of alcohol at least twice in the past 12 months
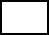
, Non-drinker (have never drunk before and those who have not drunk in the past 12 months
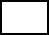


Other Substance abuse: _

1. Occupation : Employed
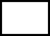
unemployed
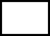

2. Marital status: Single
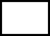
Married
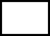


Divorced
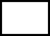
Widowed
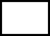


1. HIV disclosure: yes
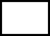
 No
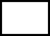

2. Monthly income (ETB)

**B.** **Laboratory Characteristic**

1. Base line CD4 (At initiation of cART)

-------cells/micro liter

1. CurrentCD4

-------cells/micro liter

1. Viralload

--------RNA/micro liter

1. HBV status: positive
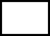
Negative
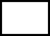
 Unknown
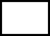

2.
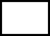

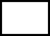
HCVstatus:positive
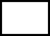
Negative Unknown

| Vital sign | BP PR RR Temp |
| --- | --- |
| CBC | WBC |
|  | RBC |
|  | Hgb |
|  | Hct |
|  | PLT |
|  | Neut |
|  | Lym |
|  | MCV |
|  | MCH |
|  | MCHC |
| LFT | ALT |
|  | AST |
|  | ALP |
| Serum albumin |  |
| RFT | Scr |
|  | bilirubin |
|  | Urine output |
| Electrolyte | Na+ |
|  | K+ |
|  | cl- |
|  | Ca2+ |

1. **Clinical characteristics**
2. Date of admission:
3. Chiefcomplaint:
4. Diagnosis atadmission
5. Clinicalpresentation(GCS)
6.
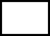
HIV sero-status: newly Diagnosed at admission
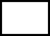
Known HIVpatient
7. If known RVI patient, Age at diagnosis Time since diagnosis in years
8. mode of acquisition
9. WHO clinical stage atdiagnosis
10. Treatment for thediagnosis
11. Number ofOIs
12.
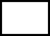
Previous history ofOIRx Yes
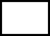
No
13. If yes, specify theOI
14. Is the patienton cART yes
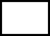
NO
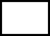

15. If yes what cART regimen:: [1e] TDF+3TC+EFV
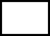
[1c]AZT+3TC+NVP
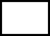


[1f]TDF+3TC+NVP
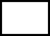
[1d]AZT+3TC+EFV
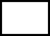


If otherspecify

1. DurationoncART_______
2.
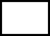
DelayedininitiationofcART?Yes
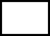
No
3. Is there any history of toxicity from cART? Yes
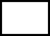
No
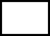

4.
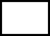
Have you ever received OIprophylaxis Yes
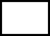
No
5. If yes, which prophylaxis CPT
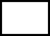
IPT
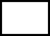
Other
6.
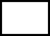
Co-morbidity Yes
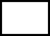
 No

If yes specify _

1. Non-invasive ventilation: yes
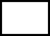
 No
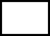

2.
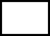
Inotropic support yes
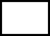
 No
3.
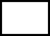

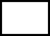
Renal replacement therapy(RRT) yes No
4. Admitted to ICU Yes
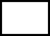
 No
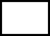

5. If yes, ICU complication AKI
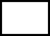
 Sepsis
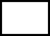
 AKI and sepsis
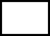

6. Outcome of Hospitalization:

Discharged with improvement
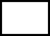
 date_______

Left against medical advice:
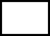
 date_________

Died:
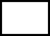
 date _______possible cause of death_________________

Lost to follow up:
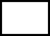
 date_______
