# Supplementary material for: Clinical outcome of admitted HIV/AIDS patients in Ethiopian tertiary care settings: A prospective cohort study
Source: PLoS One. 2019 Dec 30;14(12):e0226683. doi: 10.1371/journal.pone.0226683 (PMC6936777; doi:10.1371/journal.pone.0226683)
Supplement: S3 Dataset — (DOCX) [file pone.0226683.s003.docx]

**ANNEX-A**

**List of AIDS-related illnesses**

The current list of AIDS-defining illnesses according to the CDC are:

Bacterial infections, multiple orrecurrent

Candidiasis of bronchi, trachea, orlungs

Candidiasis of theoesophagus

Cervical cancer(invasive)

Coccidioidomycosis,disseminated

Cryptococcosis, presenting outside of thelung

Cryptosporidiosis, chronic intestinal for more than one-monthduration

Cytomegalovirus disease (other than in the liver, spleen, or lymphnodes)

Cytomegalovirus disease with loss of vision

Encephalopathy (HIV-related, also known as AIDS dementiacomplex)

Herpes simplex virus (HSV), lasting longer than a month or appearing in an areaother than the skin (such as oesophagus orlungs)

Histoplasmosis,disseminated

Kaposi's sarcoma (KS)

Lymphoid interstitial pneumonia or pulmonary lymphoid hyperplasiacomplex

Burkitt lymphoma (or equivalentterm)

Immunoblastic lymphoma (or equivalentterm)

Primary lymphoma of thebrain

Mycobacterium avium complex or Mycobacterium kansasii,disseminated

Mycobacterium tuberculosis of any site in or out of thelungs

Mycobacterium or similar species, disseminated beyond thelung

Pneumocystis pneumonia caused by the fungus Pneumocystisjiroveci

[Pneumonia](https://www.verywellhealth.com/pneumonia-overview-3156882),recurrent

[Progressive multifocal leukoencephalopathy](https://www.verywellhealth.com/progressive-multifocal-leukoencephalopathy-47943)(PML)

[Salmonella septicemia,](https://www.verywellhealth.com/salmonella-septicemia-49029)recurrent

[Toxoplasmosis](https://www.verywellhealth.com/toxoplasmosis-signs-symptoms-and-complications-4160761) of thebrain

[Tuberculosis](https://www.verywellhealth.com/tuberculosis-treatment-1958925)

[Wastingsyndrome](https://www.verywellhealth.com/hiv-wasting-syndrome-aids-defining-condition-48955)
